# Chorea as the presenting feature of acute rheumatic fever in childhood; case reports from a low-prevalence European setting

**DOI:** 10.1186/s12879-021-06005-x

**Published:** 2021-04-07

**Authors:** Marta Illán Ramos, Belén Sagastizabal Cardelús, Adrián García Ron, Sara Guillén Martín, Arantxa Berzosa Sánchez, José Tomás Ramos Amador

**Affiliations:** 1 Department of Paediatrics, Hospital Universitario Clínico San Carlos, Madrid, Spain; 2 Department of Paediatrics, Hospital Universitario de Getafe, Getafe, Madrid, Spain

**Keywords:** Acute rheumatic fever, Sydenham chorea, Hemichorea, Secondary prophylaxis, Case report

## Abstract

**Background:**

Despite a notable decrease in acute rheumatic fever (ARF) incidence in the past few decades, there are still cases in our setting. Sydenham chorea (SC) may be the initial manifestation for this condition in childhood in a significant proportion of children. We report two cases of choreoathetosis in children as the first manifestation of ARF.

**Case presentation:**

A previously healthy 8-year-old boy presented with right hemichorea with a predominance in the brachial region, orofacial dyskinesias and speech difficulties for the past 2 weeks. The only medical history of interest was a common catarrhal illness 3 weeks before and nonspecific bilateral tenosynovitis in both feet since a year prior. A brain computerized tomography was normal and the echocardiogram showed mild mitral and aortic regurgitation, meeting ARF criteria. He demonstrated clinical improvement with treatment based on prednisone and carbamazepine.

The second patient was a 10-year-old girl with choreic movements of the right half of the body and repetitive right eye closure of 1 week duration. She had symptoms of fever and rash the previous week and pharyngitis that resolved without antibiotic 2 months before. Blood tests revealed elevated C reactive protein (12 mg/dl) and erythrocyte sedimentation rate (96 mm/h). Brain magnetic resonance was normal and echocardiogram showed left ventricle dilation and mild mitral regurgitation, leading to the diagnosis of ARF. Due to neurological involvement, she received corticosteroids and intravenous immunoglobulin treatment, with worsening of neurological symptoms that required valproic acid with remission of the hemichorea. In addition skin lessions compatible with erythema marginatum appeared on the upper limbs.

**Conclusions:**

SC should be the main diagnostic consideration in cases of hemichorea with normal neuroimaging in children. The cases reported highlight the need to maintain a high index of suspicion even in settings where incidende of ARF is low and the need to perform cardiological investigations in all patients with suspected SC, due to the possibility of subclinical valve lesions. Good adherence to secondary prophylaxis is crucial to avoid chorea relapses and worsening valve disease.

## Background

Sydenham chorea (SC) represents the most common cause of acquired chorea in childhood [1, 2] and it is one of the major criteria for acute rheumatic fever (ARF) [1, 3]. SC is clearly related to *Streptococcus pyogenes* infection, and it is thought to occur as a result of an immune cross-reaction secondary to molecular mimicry leading to the binding of the antibody against immunodominant carbohydrate antigens of *S. pyogenes* to the basal ganglia [1, 3, 4].

The most common age of ARF onset is 5 to 15 years old. Most cases occur in low and middle-income countries with difficulty to determine the true incidence, mainly because regions with the highest rates are likely to have the least accurate data with substantial under-ascertainment (ie, limited access to healthcare, under-diagnosis, under-reporting) [5]. It is much less common (incidence ≤2 cases per 100.000 school-aged children) in high-income countries and rarely reported nowadays [6]. While it is not a disease of significant public health concern in many of these countries, knowledge of its features and diagnosis is still necessary given that occasional outbreaks occur, generally within indigenous and minority populations.

The higher incidence in developing countries is primarily explained by environmental factors, especially household overcrowding, which favors increased transmission of *S. pyogenes* and in smaller part by the difficulty to access to medical services [7, 8].

When ARF presents as a neurological disorder, the most frequent clinical manifestation is the appearance of SC without joint manifestations. When there is associated carditis, this is usually subclinical [9]. Chorea is a movement disorder characterised by abrupt, involuntary, irregular, purposeless, nonrhythmic, unsustained movements that flow from one part of the body to another [10] and which may be asymmetrical (hemichorea). Accompanying neuropsychiatric symptoms such as irritability, attention deficit, and obsessive-compulsive symptoms may exist. Up to 60% of patients with chorea will have residual rheumatic heart disease (persistent valvular disease) [4]. Although the vital prognosis is marked by heart disease, SC causes a significant functional repercussion on the patient [2, 3], and it may be the initial manifestation for this condition in a significant proportion.

Despite a notable decrease in ARF incidence in the past few decades in Europe [2, 3], cases are still appearing in our setting. We report two cases of choreoathetosis in children as the first clinical criteria for ARF.

## Case presentation

The first case was a Spanish 8-year-old boy (Patient 1), who presented with choreic movements for the past 2 weeks. He had a common catarrhal illness without fever 3 weeks prior and nonspecific bilateral tenosynovitis in both feet since a year ago. He had no other significant medical history, and psychomotor development was normal. He was not taking any regular medications and he lived at home with his parents and 11-years old brother. There was no history of a familial movement disorder.

On physical examination he was afebrile and haemodynamically stable and he showed right hemichorea with a predominance in the brachial region, facial grimacing and restless movements of the tongue. In addition, he demonstrated “milkmaid’s grip” associated with motor impersistence. The child seemed fidgety and inattentive and he had reduced verbal fluency and executive functions. He did not show compulsions or obsessional symptoms, but he had emotional lability.

Cardiopulmonary auscultation and the rest of the physical examination were normal except for bilateral ankle edema without limitation of movement or other inflammatory signs.

An urgent brain computerized tomography (CT) without contrast ruled out bleeding and signs of intracranial hypertension, with a normal white and grey brain matters differentiation. Blood tests were performed (Table 1) with no significant abnormalities (6100 leukocytes/mm^3^, C reactive protein (CRP) 1.1 mg/dL and erythrocyte sedimentation rate (ESR) 20 mm/h). He was admitted for further work up.

**Table 1:** Complementary studies performed in patients at hospital admission

COMPLEMENTARY STUDY	PATIENT 1 (8 years old)	PATIENT 2 (10 years old)
**Blood test**	Normal results for: blood count (hemoglobin 12.9 mg/dL, leukocytes 6300/mm^3^, platelets 302,000/mm^3^), biochemistry, liver enzymes, folic acid, vitamin B12, creatine kinase, ammonium, homocisteyne, copper, ceruloplasmin and thyroid hormones. CRP 1.1 mg/dL, ESR 20 mm/h, Anti-streptolysin O 458 IU/mL (ULN for age 265 IU/mL) [10]	Normal results for: blood count (hemoglobin 12.8 mg/dL, platelets 318,000/ mm^3^) except leucocytes 16,010/mm^3^ and 70% neutrophils, biochemistry, liver enzymes, copper, ceruloplasmin and thyroid hormones. CRP 12 mg/dL, ESR 96 mm/h, Anti-streptolysin O 956 IU/Ml (ULN for age 276 IU/mL) [10], Anti DNAse B > 1600 IU/mL (ULN for age 499 IU/mL) [10]
**Auto-antibodies**	ANA, anti-transglutaminase antibodies, anti-gliadin antibodies, anti-phospholipid antibodies, anti-thyroid antibodies: negative.	ANA, anti-double stranded DNA antibodies, rheumatoid factor: negative
**Cerebrospinal fluid (CSF) test**	Clear and colourless, red blood cells 0/μL, leukocytes 4/μL, protein 61 mg/dL, glucose 16 mg/dL	
**Neurotropic virus polymerase chain reaction in CSF**	Varicella-zoster virus, herpes simplex 1 and 2 virus, enterovirus, Epstein-Barr virus, citomegalovirus, human herpesvirus 6, 7 and 8: negative	
**Electroencephalogram**	Normal	Normal
**Cranial computerized tomography without contrast**	Normal	Not interpretable because of the chorea movements
**Brain magnetic resonance imaging (with and without contrast)**	No anatomic alterations. Ventricular system with normal morphology and size. There are no white matter signal intensity alterations suggesting pathology. No restriction areas are observed in diffusion sequences.	Anatomy of supratentorial structures and posterior fossa are normal. Normal craniocervical junction. Ventricular system with normal morphology and size. There are no white matter signal intensity alterations suggesting pathology. No restriction areas are observed in diffusion sequences.
***S. pyogenes*** **pharyngeal culture**	Negative	Negative
**Chest x-ray**	Not performed	Normal
**Electrocardiogram**	Normal (PR 130 ms, no block heart)	Normal sinus rhythm, heart rate 100 bpm. QRS axis 75°. PR interval 136 ms. QTc: 440 ms. No hypertrophy signs.
**Echocardiogram**	Leaflet thickening anterior mitral valve with mild mitral insufficiency and mitral valve prolapse. Aortic valve with 16.7 mm diameter of the aortic annulus and thickening resulting in mild aortic insufficiency.	Mild-moderate mitral insufficiency. Dilation of the left ventricle.

*ANA* antinuclear anti-body, *CSF* cerebrospinal fluid, *CRP* C reactive protein, *ESR* erythrocyte sedimentation rate, *ULN* upper limit of normal

Cerebrospinal fluid test and blood panel antibodies (anti-nuclear (ANA), anti-transglutaminase, anti-gliadin, anti-phospholipid, anti-thyroid) were normal (Table 1). Other blood tests such as serum copper and ceruloplasmin were also negative. Brain magnetic resonance imaging (MRI) did not reveal anormal findings (Table 1).

Electrocardiogram (ECG) was normal (PR Interval 130 ms and no heart block), and the echocardiogram showed mild aortic regurgitation and a thickening in the valve’s anterior leaflet of mitral valve causing mild mitral regurgitation (Table 1), meeting ARF criteria. Although streptococcal pharyngeal culture was negative, the antistreptolysin O titer (ASOT) decrease from 458 IU/mL at diagnosis to 291 IU/mL in 4 weeks with gradual normalization subsequently.

Prednisone (2 mg/kg/day for 2 weeks with gradual tapering, over a total of 6 weeks of therapy) and carbamazepine (17 mg/kg/day) treatment were started. The boy demonstrated a very favorable evolution, with complete movement disorder resolution after 1 month, except for occasional mild difficulty in speech. After 2 months of carbamazepine treatment, it was discontinued.

A relapse of chorea generalized to both sides (with negative *S. pyogenes* pharyngeal culture) was observed 6 months after the initial presentation, that subsided with reintroduction of carbamazepine treatment with a complete neurological recovery in 2 weeks. Carbamazepine was continued for ten months without further clinical recurrence after stopped therapy over 18 months of follow-up.

The second case was a 10-year-old girl (Patient 2) with right limb and hemifacial choreic movements of 1 week duration. She had symptoms of fever and papular and not confluent rash the previous week and a history of pharyngitis that resolved without antibiotic treatment 2 months before. She was a previously healthy Spanish girl, with no significant medical history and without regular medications. She lived with her parents and her sister. There was no history of a familial movement disorder.

On physical examination she was afebrile and with normal vital signs. She presented choreic movements of the right half of the body, involuntary lingual movements, and repetitive right eye closure. No murmurs or rubs were detected on the cardiopulmonary auscultation. In addition, she exhibited skin lessions compatible with erythema marginatum on the upper limbs the third day of hospital admission.

Blood laboratory tests (Table 1) demonstrated elevation of acute phase reactants (CRP 12 mg/dl and ESR 96 mm/h, leukocytes 16,010/mm^3^) with normal serum copper, ceruloplasmin and ANA. Streptococcal pharyngeal culture was negative with a high ASOT (956 IU/mL) and anti-DNAse B titres (> 1600 IU/mL). The ASOT peaked at 1060 IU/mL at day 12, with subsequent decrease and normalisation over 2 months.

Brain CT was performed but not valid because of the patient’s involuntary movements, and posterior brain MRI was considered normal (Table 1).

Chest x-ray did not reveal cardiomegaly or other pathological findings. Initial ECG was normal and the echocardiogram showed left ventricle dilation and mild mitral regurgitation (Table 1), fulfilling criteria for the diagnosis of ARF. During follow-up cardiological workup, first-degree AV block was observed in the ECG, and mild mitral and aortic regurgitation was noted on echocardiography.

Due to neurological involvement, intravenous immunoglobulin treatment (400 mg/kg/day) was given for 5 days, with worsening of neurological symptoms associated with dyskinesia in the left half of the body and involuntary jerky movements that required initiation of valproic acid. She received treatment with corticosteroids (2 mg/kg/day) for 2 weeks followed by gradual tapering that was complete in 6 weeks from the start, with improvement of symptoms.

Neurologically she was completely recovered at 3 weeks and at this time she does not receive any antiepileptic medication (valproic acid was given during 2 months). During the 8 subsequent years she has not presented new ARF recurrences.

Both patients received treatment against *S. pyogenes* with oral penicillin for 10 days and subsequently initiated secondary prophylaxis with oral penicillin V twice daily because they and their parents preferred this option rather than the intramuscular regimen every 4 weeks. Adherence to oral prophylaxis been good during follow-up (over a year and a half in patient 1 and 8 years in patient 2 and still continue today in either way).

## Discussion and conclusions

Our two cases provide evidence of the persistence of ARF in Spain, even though a dramatic decrease in the incidence has been observed in the last decades. Unlike other major ARF criteria, SC usually occurs after a longer latent period with intervals of 1 to 8 months following streptococcal infection [1, 4]. Thus it is uncommon to identify *S. pyogenes* from throat culture, so in addition blood tests for streptococcal antibodies should be obtained to assess for preceding streptococcal infection. Because of this and the possible onset of chorea as the only overt manifestation of ARF, initial diagnosis may be difficult and always it is necessary to exclude alternative aetiologies of acquired chorea: autoimmune or inflammatory, cerebrovascular, drugs, infections, metabolic disorders or neoplasia.

Patient 1 did not have a clear streptococcal infection antecedent, however in the absence of other findings in the movement disorders study with a raised ASOT level [11], and the mitral and aortic insufficiency, two major Jones criteria were met.

Clinical carditis has classically been defined as an audible murmur consistent with aortic or mitral regurgitation. In the past few years, developments in several areas have prompted reexamination of the traditional Jones criteria [12]. The current ones include subclinical carditis like one of the major criteria [12, 13] after numerous studies have addressed the role of echocardiography in the diagnosis of ARF and have reported echocardiography/Doppler evidence of mitral or aortic valve regurgitation in patients with ARF despite the absence of classic auscultatory findings. In our cases both patients met criteria for ARF with 2 major criteria (chorea and subclinical carditis).

Chorea associated with ARF in children usually has a self-limited clinical course lasting 5–16 weeks. It commonly starts as a hemichorea (30%) [1, 2]. It may also follow a fluctuating course over many months, even years, before eventually resolving, and some patients like the children reported may need treatment to control neurologic symptoms. Nevertheless, the prognosis generally is good with complete recovery in most cases.

Our cases with negative pharyngeal culture reflect that since ARF is a “post-streptococcal” phenomenon and bacterial culture evidence of streptococcal infection in the throat is usually absent at the time of diagnosis. Regardless of the pharyngeal culture result, initial antibiotic treatment is indicated [3, 4, 13].

Secondary prophylaxis is indicated in all cases of ARF, irrespective of whether isolated SC or cases with heart disease are identified. Subsequent adherence to secondary prophylaxis is absolutely crucial to avoid chorea relapses and valve disease worsening [1, 4]. In this respect, although the preferred option is intramuscular penicillin G benzathine every 3–4 weeks because it facilitates therapeutic compliance, daily oral penicillin V is also considered an alternative regimen for patients or families who refuse the regular injection schedule [3, 13, 14]. In our cases, the chosen option was the oral regimen due to the patients´ preferences and the families’easy accesibility to healthcare. We offered the option of changing to intramuscular regime in the future. The follow-up was done in cooperation between Pediatrics Infectious Diseases and Primary Care.

The duration of prophylaxis depends on the presence of carditis and associated valvular disease, varying from 5 years in ARF without carditis to 10 years or until 21 years of age (whichever is longer) in carditis without residual heart disease, and 10 years or until 40 years of age (whichever is longer) if there is persistent valvular disease, according to the last American Academy of Pediatrics recommendations [13]. By following these recommendations, in our patients, due to the residual valvular insufficiency, it is planned to continue prophylaxis until 40 years of age.

The cases reported received different therapeutic managemet of the neurological disorder, but both underscore the need to maintain a high index of suspicion of ARF even in settings where its current incidence is very low. In addition, they highlight the need for immediate cardiological investigations in all patients with suspected SC, due to the possibility of subclinical valve lesions, which determines the patient management and their prognosis. SC should be the main diagnostic consideration in cases of hemichorea with normal neuroimaging in children, even in settings with low incidence of ARF.

## Data Availability

Data sharing is not applicable to this article as no datasets were generated or analysed.

## Abbreviations

SC: Sydenham chorea
ARF: Acute rheumatic fever
CRP: C reactive protein
ESR: Erythrocyte sedimentation rate
ANA: Antinuclear antibody
CT: Computerized tomography
MRI: Magnetic resonance imaging
ECG: Electrocardiogram
ASOT: Antistreptolysin O titer

## References

[1] García González MM, Mayol Canals L, Villalobos Arévalo P, Vázquez Ruiz M, Cabacas GA (2007). Corea de Sydenham: presentación de un caso tratado con carbamazepina con excelente respuesta clínica. An Pediatr (Barc).

[2] Fernández Ávalos S, Claret Teruel G, González Álvareza G, Luaces CC (2008). Corea de Sydenham: un pasado aún presente. An Pediatr (Barc).

[3] Walker KG, Wilmshurst JM (2010). An update on the treatment of Sydenham’s chorea: the evidence for established and evolving interventions. Ther Adv Neurol Disord.

[4] Webb RH, Grant C, Harnden A (2015). Acute rheumatic fever. BMJ..

[5] Noonan S, Zurynski YA, Currie BJ, McDonald M, Wheaton G, Nissen M (2013). A national prospective surveillance study of acute rheumatic fever in Australian children. Pediatr Infect Dis J.

[6] Miyake CY, Gauvreau K, Tani LY, Sundel RP, Newburger JW (2007). Characteristics of children discharged from hospitals in the United States in 2000 with the diagnosis of acute rheumatic fever. Pediatrics..

[7] Jaine R, Baker M, Venugopal K (2011). Acute rheumatic fever associated with household crowding in a developed country. Pediatr Infect Dis J.

[8] Carapetis JR, Beaton A, Cunningham MW, Guilherme L, Karthikeyan G, Mayosi BM, Sable C, Steer A, Wilson N, Wyber R, Zühlke L (2016). Acute rheumatic fever and rheumatic heart disease. Nat Rev Dis Primers.

[9] Krishna Kumar R, Tandon R (2013). Rheumatic fever & rheumatic heart disease: the last 50 years. Indian J Med Res.

[10] Crealey M, Allen NM, Webb D, Bouldin A, Mc Sweeney M, Peake D (2015). Sydenham's chorea: not gone but perhaps forgotten. Arch Dis Child.

[11] Steer AC, Vidmar S, Ritika R, Kado J, Batzloff M, Jenney AWJ, Carlin JB, Carapetis JR (2009). Normal ranges of streptococcal antibody titers are similar whether streptococci are endemic to the setting or not. Clin Vaccine Immunol.

[12] Gewitz MH, Baltimore RS, Tani LY, Sable CA, Shulman ST, Carapetis J, Remenyi B, Taubert KA, Bolger AF, Beerman L, Mayosi BM, Beaton A, Pandian NG, Kaplan EL, American Heart Association Committee on Rheumatic Fever, Endocarditis, and Kawasaki Disease of the Council on Cardiovascular Disease in the Young (2015). Revision of the Jones criteria for the diagnosis of acute rheumatic fever in the era of Doppler echocardiography: a scientific statement from the American Heart Association. Circulation.

[13] American Academy of Pediatrics. Group A Streptococcal Infections. In: Kimberlin. DW, Brady MT, Jackson MA, Long SS, editors. Red Book: 2018 Report of the committee on infectious diseases, vol. 2018. 31st ed. Itasca, IL: American Academy of Pediatrics. p. 757–61.

[14] WHO Study Group on Rheumatic Fever and Rheumatic Heart Disease (2001: Geneva, Switzerland) & World Health Organization. Rheumatic fever and rheumatic heart disease: report of a WHO expert consultation, Geneva, 20 October-1 November 2001: World Health Organization; 2004. https://apps.who.int/iris/handle/10665/42898

